# Supplementary material for: Verbascoside Elicits Its Beneficial Effects by Enhancing Mitochondrial Spare Respiratory Capacity and the Nrf2/HO-1 Mediated Antioxidant System in a Murine Skeletal Muscle Cell Line
Source: Int J Mol Sci. 2023 Oct 17;24(20):15276. doi: 10.3390/ijms242015276 (PMC10607197; doi:10.3390/ijms242015276)
Supplement: Supplementary file 1 [file ijms-24-15276-s001.zip › ijms-2628548-supplementary.pdf]

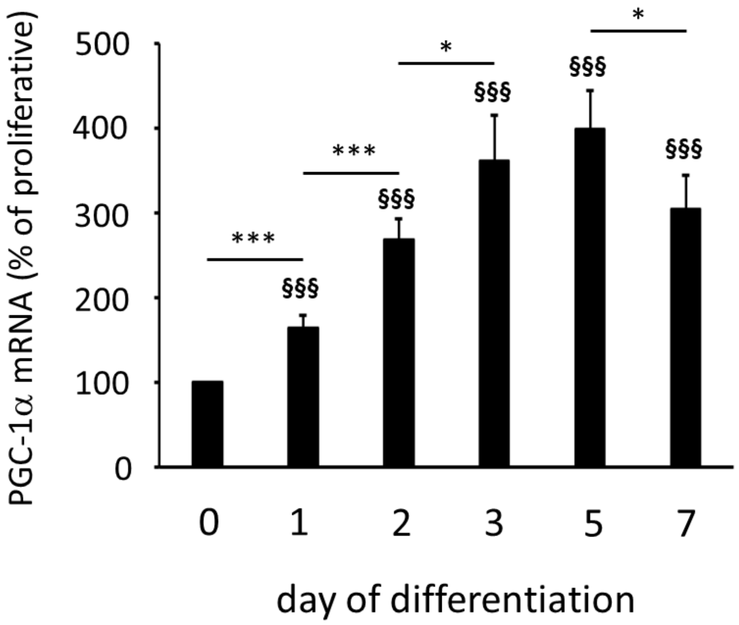

Figure S1 Transcription of PGC-1α gene during C2C12 differentiation. mRNA levels were evaluated by Real-Rime PCR and are expressed as the mean percentage relative to cells in proliferative conditions (control 100%) ± SD of three independent experiments run in triplicate. Differences between mean values were assessed by Student's t-test (* $p \leq 0.05$, *** $p \leq 0.001$; §§§ $p \leq 0.001$ differentiated cells vs control).
